# Interaction of Synthetic Human SLURP-1 with the Nicotinic Acetylcholine Receptors

**DOI:** 10.1038/s41598-017-16809-0

**Published:** 2017-11-30

**Authors:** Thomas Durek, Irina V. Shelukhina, Han-Shen Tae, Panumart Thongyoo, Ekaterina N. Spirova, Denis S. Kudryavtsev, Igor E. Kasheverov, Grazyna Faure, Pierre-Jean Corringer, David J. Craik, David J. Adams, Victor I. Tsetlin

**Affiliations:** 1 Institute for Molecular Bioscience, The University of Queensland, Brisbane, QLD 4072 Australia; 2 Shemyakin-Ovchinnikov Institute of Bioorganic Chemistry, Russian Academy of Sciences, Moscow, 117997 Russia; 3 Illawarra Health and Medical Research Institute (IHMRI), University of Wollongong, Wollongong, NSW 2522 Australia; 4 Channel-Receptors Unit, Institut Pasteur, 75015 Paris, France; CNRS UMR 3571, 75015 Paris, France; 5 Present Address: Faculty of Science and Technology, Thammasat University, Bangkok, Thailand

## Abstract

Human SLURP-1 is a secreted protein of the Ly6/uPAR/three-finger neurotoxin family that co-localizes with nicotinic acetylcholine receptors (nAChRs) and modulates their functions. Conflicting biological activities of SLURP-1 at various nAChR subtypes have been based on heterologously produced SLURP-1 containing N- and/or C-terminal extensions. Here, we report the chemical synthesis of the 81 amino acid residue human SLURP-1 protein, characterization of its 3D structure by NMR, and its biological activity at nAChR subtypes. Radioligand assays indicated that synthetic SLURP-1 did not compete with [^125^I]-α-bungarotoxin (α-Bgt) binding to human neuronal α7 and *Torpedo californica* muscle-type nAChRs, nor to mollusk acetylcholine binding proteins (AChBP). Inhibition of human α7-mediated currents only occurred in the presence of the allosteric modulator PNU120596. In contrast, we observed robust SLURP-1 mediated inhibition of human α3β4, α4β4, α3β2 nAChRs, as well as human and rat α9α10 nAChRs. SLURP-1 inhibition of α9α10 nAChRs was accentuated at higher ACh concentrations, indicating an allosteric binding mechanism. Our results are discussed in the context of recent studies on heterologously produced SLURP-1 and indicate that N-terminal extensions of SLURP-1 may affect its activity and selectivity on its targets. In this respect, synthetic SLURP-1 appears to be a better probe for structure-function studies.

## Introduction

**Figure 1:**
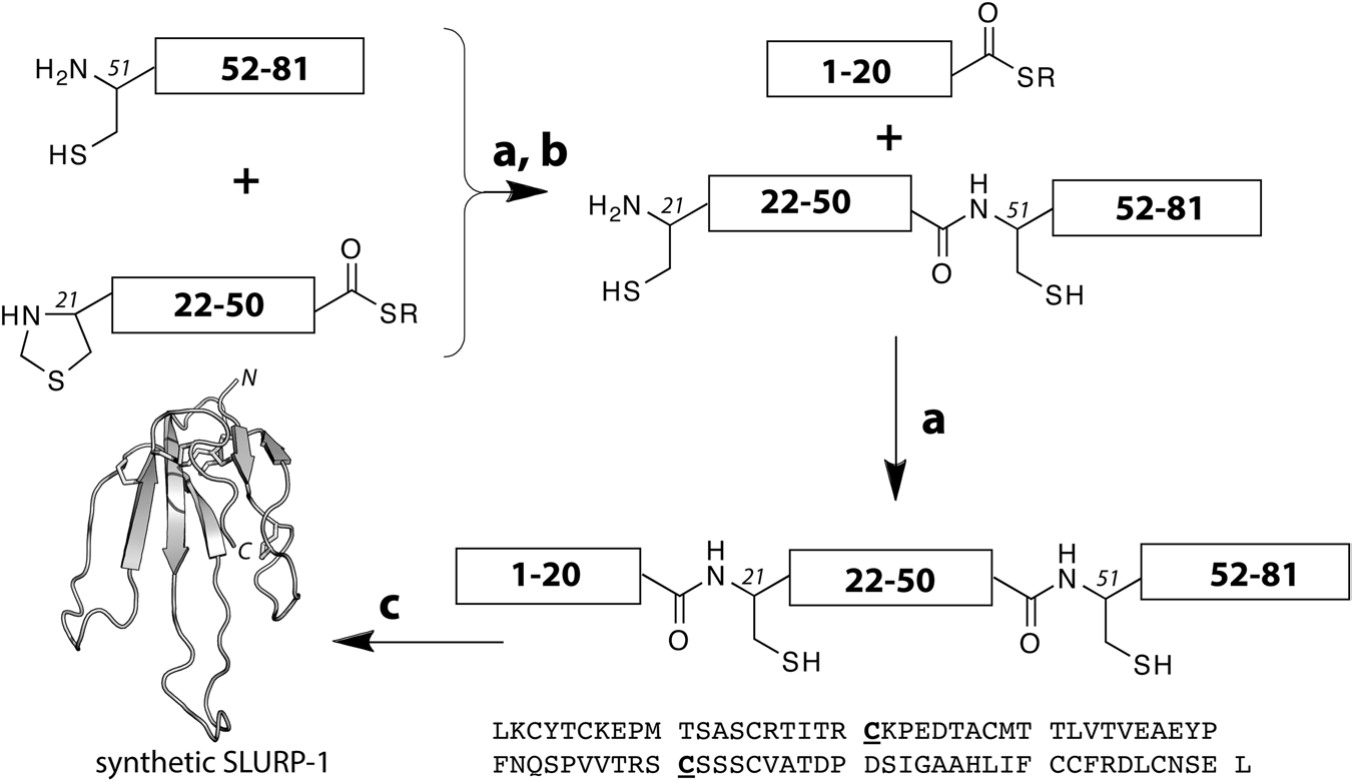
Chemical synthesis of SLURP-1 via one-pot native chemical ligation (NCL). (**a**) NCL, (**b**) Thz to Cys conversion and (**c**) folding and disulfide bond formation. The primary structure and sequence^5^ of human SLURP-1 are shown at the bottom. The two cysteines that were used as ligation sites are highlighted.

The three-finger fold is a protein domain structure comprising a disulfide-stabilized core from which three elongated loops (fingers) protrude (Fig. 1). It features prominently in two large protein families: snake venom neurotoxins and the Ly6 proteins, the latter first discovered in the mammalian immune system^1–4^. Besides their similar 3D structures, proteins with this fold also share a similar genetic organization and a conserved pattern and connectivity of cysteine residues that ultimately form the structure-stabilizing disulfides. These common features provide strong evidence that Ly6 proteins and snake venom neurotoxins are evolutionary related, however, despite the structural similarities the functional link between these two families has only emerged recently.

Most Ly6 proteins are membrane-tethered by a covalently attached glycosyl phosphatidylinositol (GPI) anchor, such as for Ly6/neurotoxin 1 (Lynx1), but some are secreted proteins including SLURP-1 (**s**ecreted **L**y6/**u**rokinase-type plasminogen **r**eceptor-related **p**rotein), which was initially isolated from human blood and urine^5^. SLURP-1 is also expressed in keratinocytes and *SLURP-1* mutations are implicated in the Mal de Meleda skin disease^4,6^. Additionally, SLURP-1 has been reported to regulate processes in the immune and nervous systems^7–9^.

SLURP-1 (as well as other Ly6 proteins such as Lynx1 and SLURP-2) represents a functional link between the mammalian Ly6 proteins and snake neurotoxins. Many members from the latter group, which include the well characterized pharmacological agents α-bungarotoxin (α-Bgt) and α-cobratoxin (α-Cbt), are potent inhibitors of nicotinic acetylcholine receptors (nAChR). Co-localization studies and functional *in vitro* activity data have demonstrated that certain Ly6 proteins (Lynx1 and SLURP-1 and -2) also interact with nAChRs, suggesting that they might function as endogenous modulators of nAChR signaling *in vivo* ^10,11^.

Various recombinant versions of SLURP-1 have been shown to modulate nAChRs, mostly of the α7 subtype^7,8,12^, but with contradictory results due to the expressed SLURP-1 proteins containing additional C- or N-terminal fusion tags. For example, Chimienti and colleagues reported potentiation of α7 nAChR-mediated currents by a recombinant myc-His_6_-SLURP-1 fusion construct at low nanomolar concentrations^12^. In contrast, recombinant SLURP-1 expressed in *E. coli*, containing an additional methionine residue at the N-terminus (hereafter referred to as rSLURP-1), exhibited inhibitory activity at α7 nAChR (at micromolar concentrations)^13^.

To resolve these conflicting data on SLURP-1 activities, we report here the chemical synthesis and biological activity of the 81 amino acid human SLURP-1 identical in amino acid sequence to the human serum-derived protein. Using a combination of solid phase peptide synthesis and native chemical ligation^14^, high purity protein was obtained in multi-milligram amounts sufficient for structural and functional studies. The synthetic protein was characterized by HPLC, MS, and NMR which confirmed the three-finger fold structure. Most importantly, our pharmacological data revealed for the first time the interaction of synthetic SLURP-1 (sSLURP-1) with several neuronal nAChR subtypes.

## Results

### Human SLURP-1 synthesis and NMR structural analysis

**Figure 2:**
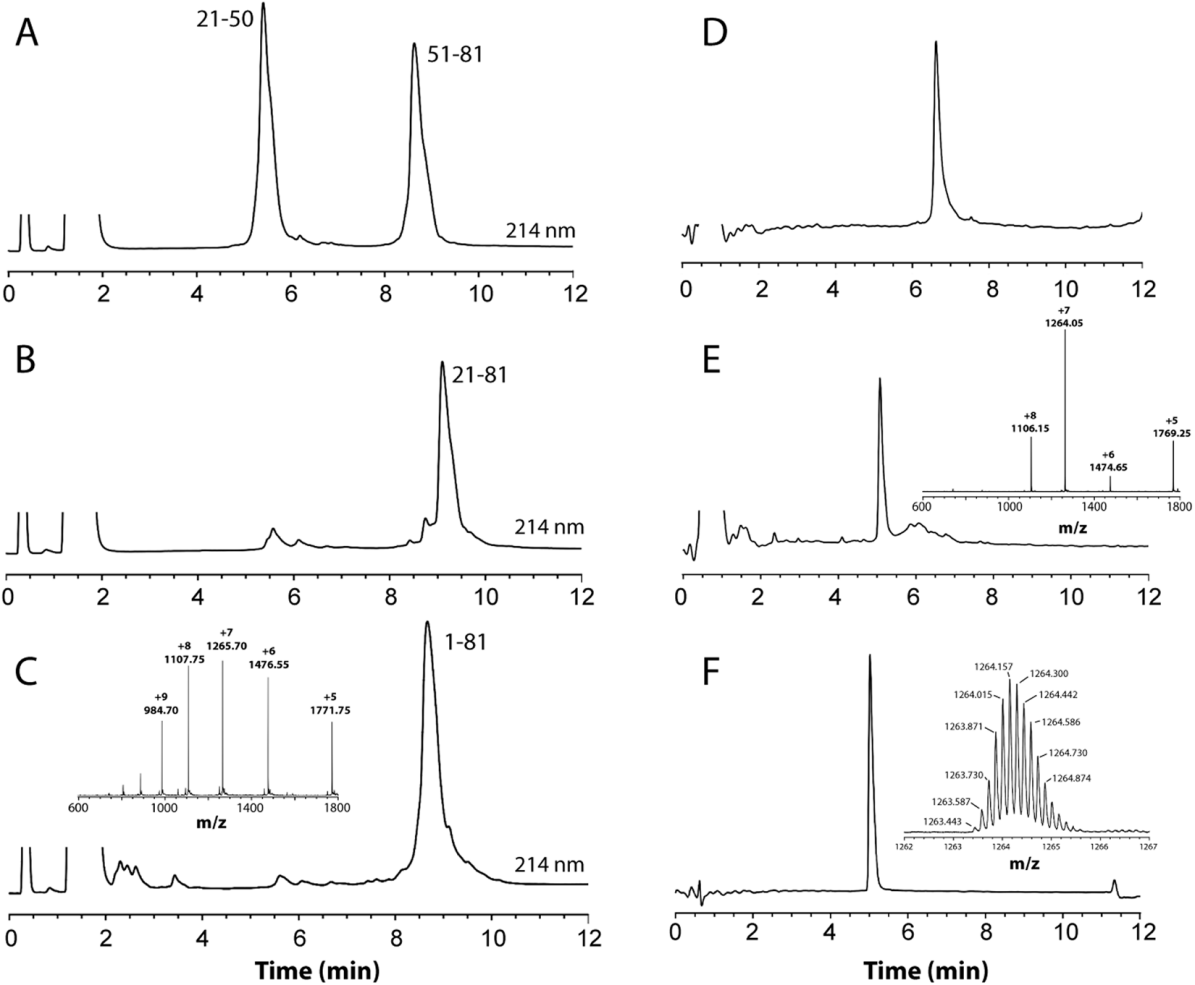
Monitoring chemical synthesis of SLURP-1 by uHPLC. (A-C) One-pot NCL of SLURP-1 segments. (**A**) Ligation of SLURP-1[21–50] and SLURP-1[51–81]-α-thioester segments at t = 0 and, (**B**) after 12 h ligation and Thz to Cys conversion. (**C**) SLURP-1[1–20]-α-thioester segment was subsequently added and ligated to fragment 21–81 to form the reduced SLURP-1 polypeptide. (**D,E**) Folding of the SLURP-1 polypeptide monitored by uHPLC. (**D**) Reduced SLURP-1 and (**E**) crude folding mixture after 70 h. The principal peak at retention time 5 min corresponds to correctly folded human SLURP-1. ESI-MS spectra of the dominant peaks are shown as inserts. (**F**) uHPLC and high-resolution MS analysis of purified human sSLURP-1. The inset shows an isotopically resolved blow-up of the [M + 7 H]^7+^ charge state.

Human SLURP-1, with 81 amino acid residues and five disulfide bonds, is considerably larger than most three-finger proteins from snake venoms (up to 62 residues and four disulfides). It is reminiscent of the classical long-chain α-neurotoxins, which are typically composed of up to 75 residues with a 5^th^ disulfide bond in the central loop II. In contrast, in all Ly6 proteins, including SLURP-1, the 5^th^ disulfide resides in the N-terminal loop I^4^. Given the size of the target SLURP-1 molecule, we resorted to a peptide segment ligation approach to overcome the size limitation of traditional stepwise solid phase peptide synthesis (SPPS) (Figs 1 and 2)^15^. Accordingly, the SLURP-1 polypeptide chain was split into three segments, which were individually assembled by either Boc or Fmoc SPPS (see Materials and Methods). Thiazolidine-4-carboxylic acid (Thz) was used in place of Cys21^16,17^ to prevent cyclisation and oligomerisation during the first chemical ligation of SLURP-1[21–50] and SLURP-1[51–81]. Following cleavage from the solid support and purification, the segments were joined via native chemical ligation in one-pot fashion as described previously^16,18^.

The fully reduced SLURP-1 polypeptide was obtained in good yield (69% based on the limiting starting peptide segment SLURP-1[51–81]). Folding and disulfide formation of the synthetic molecule was achieved using protocols described recently for inclusion body refolding of rSLURP-1 produced in *E. coli* ^13,19^. The folding kinetics and the overall HPLC folding profile were essentially identical to those reported for rSLURP-1 and allowed preparation of synthetic SLURP-1 in high purity and in multi-milligram quantities (Fig. 2D–F). High resolution MS analysis indicated a monoisotopic mass of 8837.1 ± 0.1 Da, in excellent agreement with the theoretical monoisotopic mass of 8837.02 Da demonstrating formation of five disulfide bonds (Fig. 2F).

**Figure 3:**
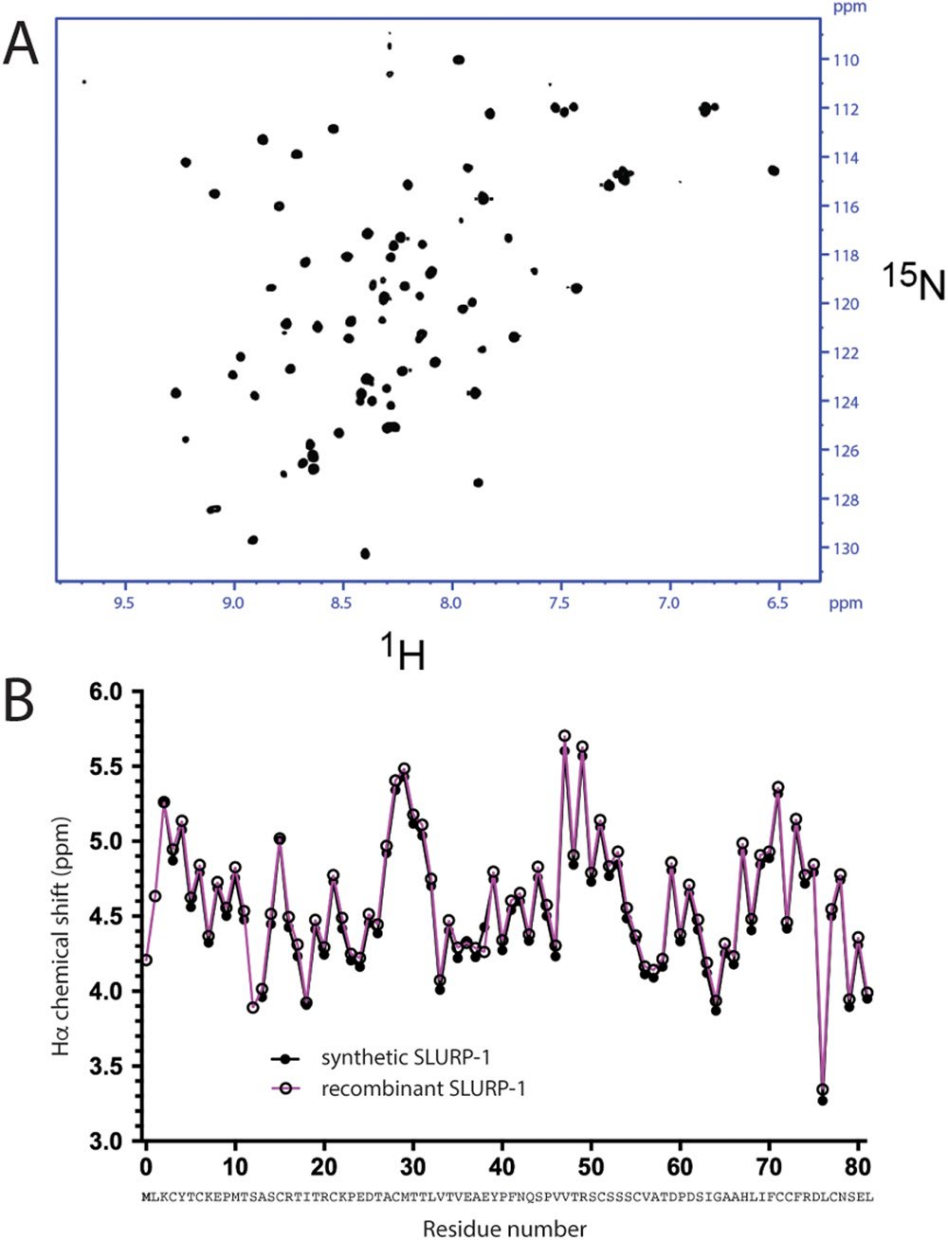
NMR characterization of synthetic human SLURP-1. (**A**) ^1^H-^15^N HSQC spectrum of synthetic SLURP-1 (H_2_O/D_2_O (9:1), pH 4.8, 310 K) and (**B**) Hα chemical shift comparison of synthetic SLURP-1 and rSLURP-1 (BMRB 25225, PDB ID: 2MUO).

To verify the anticipated three-finger fold of the synthetic material, we performed NMR experiments under the same conditions as reported previously for rSLURP-1 (i.e., H_2_O/D_2_O (9:1), pH 4.8, 310 K)^19^. The natural abundance ^1^H-^15^N HSQC spectrum of SLURP-1 showed good dispersion of amide proton and nitrogen resonances (Fig. 3A) suggesting that the molecule adopts a well-defined three-dimensional structure. Overall, the spectrum is highly similar to that of rSLURP-1^19^. Two-dimensional TOCSY and NOESY spectra were used to assign backbone amide and CαH protons. Comparison of the Hα chemical shift of each residue obtained from synthetic SLURP-1 spectra to the values available for rSLURP-1 (BMRB ID: 25225 and 25226; Fig. 3B) revealed excellent agreement, suggesting the proteins have highly similar three-dimensional structures. This suggestion is further supported by several key long-range NOEs observed in the NOESY spectra of synthetic SLURP-1, including Lys2Hα-Arg20Hα, Cys28Hα-Cys51Hα, Met29Hα-Cys71Hα, Thr30Hα-Arg49Hα and Leu76Hα-Tyr4Hδ/ε, all consistent with the proposed three-finger fold. Taken together, these data confirm that our synthetic SLURP-1 has a tertiary structure similar to that of rSLURP-1.

### Human SLURP-1 does not compete with α-Bgt at human neuronal α7, muscle-type nAChRs and AChBPs

**Figure 4:**
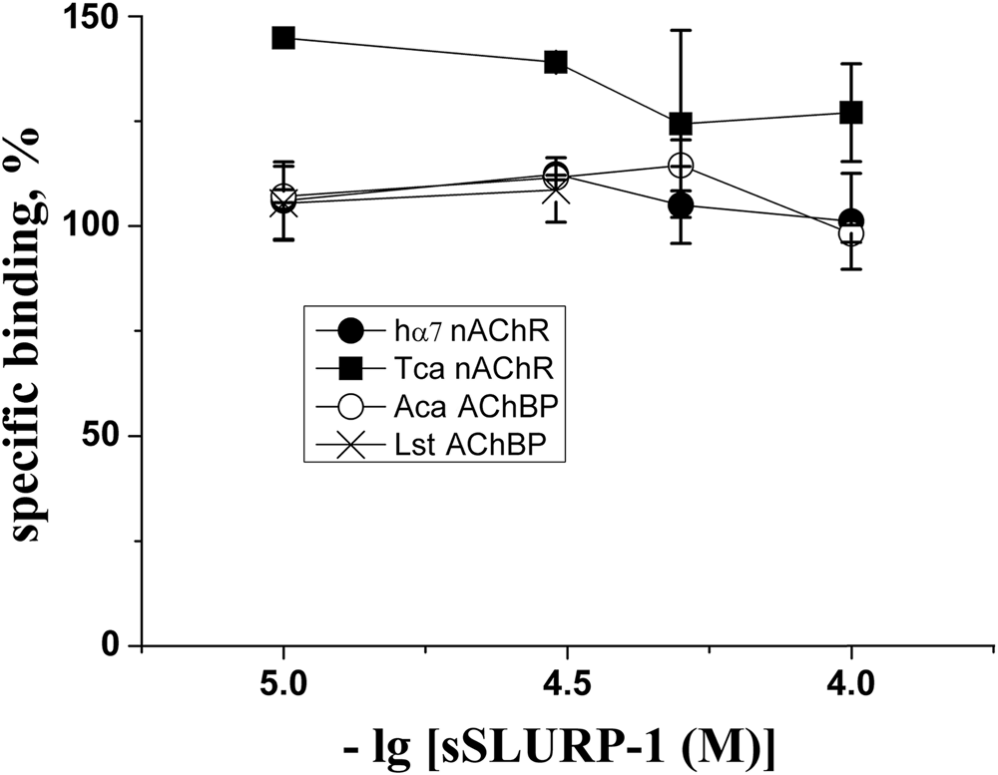
Synthetic SLURP-1 activity at the orthosteric site of human α7 nAChR expressed in GH_4_C_1_ cells, muscle-type nAChR of *T. californica* (Tca), and AChBPs from *A. californica* (Aca) and *L. stagnalis* (Lst). Binding was assessed in competition with [^125^I]-α-Bgt (0.2 nM). Each data point represents the mean ± SEM of 3 independent experiments.

The recombinant form of SLURP-1 was shown previously to displace bound α-Bgt from the muscle-type nAChR of *Torpedo californica* and the mollusk *Lymnaea stagnalis* AChBP^13^. However, in the present study, the synthetic version of SLURP-1 did not compete with α-Bgt for either proteins (Fig. 4). In addition, no competition with α-Bgt binding was observed at either human (h) α7 nAChR or *Aplysia californica* AChBP (Fig. 4).

### SLURP-1 inhibition of hα7 nAChR in the presence of the positive allosteric modulator PNU120596

**Figure 5:**
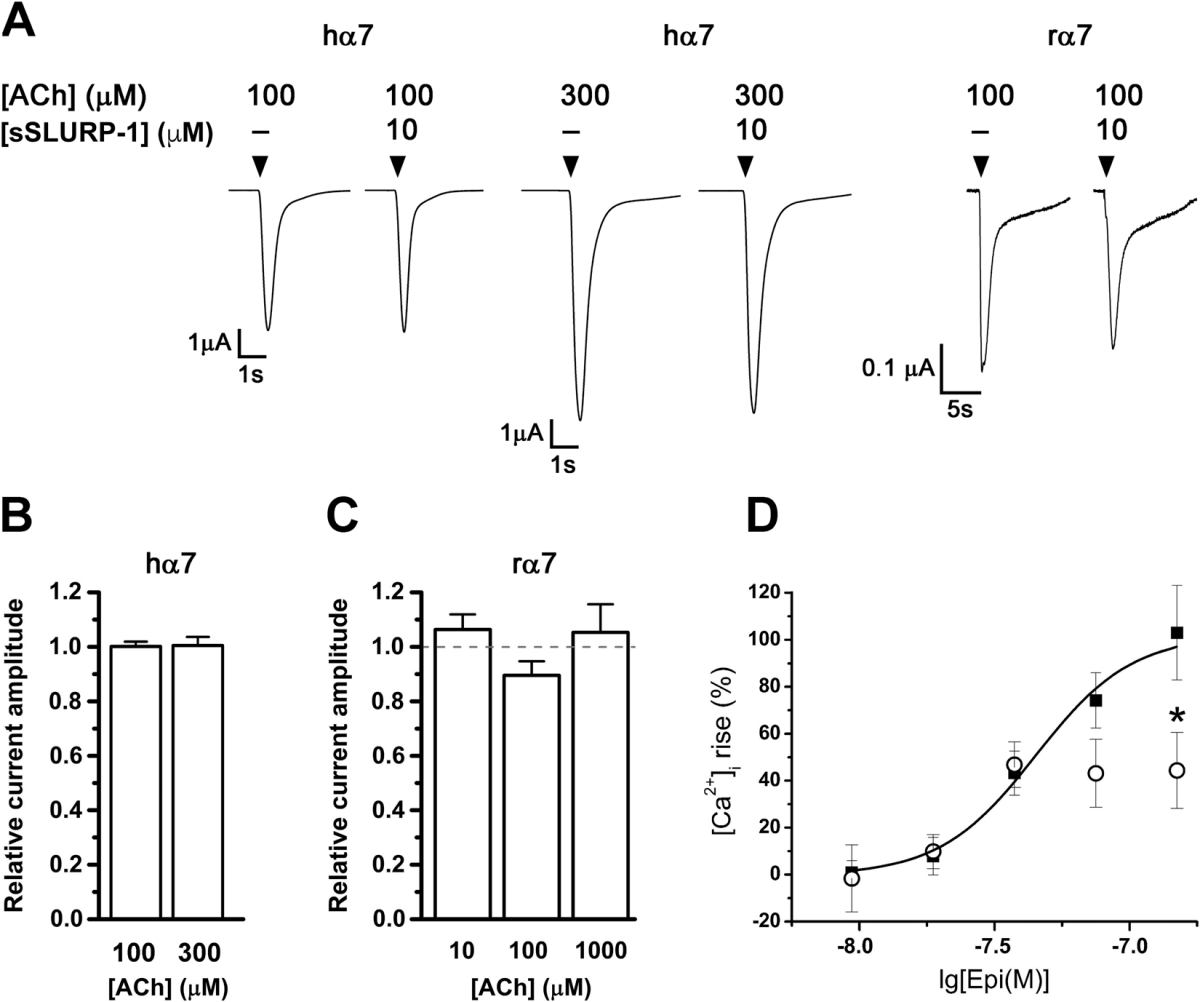
Activity of synthetic SLURP-1 on agonist-evoked response mediated by α7 nAChRs. (**A**) Representative ACh-evoked current traces through hα7 and rα7 nAChRs in the presence of 10 µM sSLURP-1. (**B**) Bar graph of sSLURP-1 activity (10 μM) on ACh-evoked peak current amplitude mediated by hα7 and (**C**) rα7 nAChRs expressed in *X. laevis* oocytes. (**D**) Concentration-response curve of epibatidine (Epi) + 10 µM PNU120596-induced intracellular calcium ion concentration ([Ca^2+^]_i_) rise in Neuro2a cells expressing hα7 nAChRs in the absence (*black squares*, EC_50_ = 44.9 ± 4.6 nM) and presence of 5 µM sSLURP-1 (*open circles*). Mean ± SEM, n = 3–10. *P < 0.05 vs [Ca^2+^]_i_ rise evoked by corresponding agonist concentration in the absence of sSLURP-1, unpaired two-tailed Student’s t-test.

In the [^125^I]-α-Bgt binding assay, synthetic SLURP-1 showed no competitive antagonism at hα7 nAChR, consistent with the reported inactivity of rSLURP-1 in the same assay^13^. However, rSLURP-1 inhibited ACh-evoked currents at hα7 nAChR^13^ and since the inhibition showed a direct relationship with the ACh concentration, we tested synthetic SLURP-1 at hα7 and rat (r) α7 nAChRs heterologously expressed in *Xenopus laevis* oocytes, under similar conditions (Fig. 5). At 10 μM, regardless of the ACh concentrations used (10, 100, 300, or 1000 µM), synthetic SLURP-1 did not antagonize ACh-evoked currents mediated by hα7 (Fig. 5A,B) and rα7 (Fig. 5A and C) nAChR subtypes.

The activity of some ligands at α7 nAChR-mediated currents can be amplified in the presence of the α7 subtype specific positive allosteric modulator PNU120596^20^. Therefore, we investigated the activity of sSLURP-1 in the presence of 10 μM PNU120596 and indeed, inhibition of epibatidine (Epi)-induced Ca^2+^ influx (59% inhibition by 5 μM SLURP-1 at 150 nM Epi) was observed in neuroblastoma Neuro2a cells expressing hα7 nAChR (Fig. 5D). sSLURP-1 was also tested on mouse muscle nAChRs expressed in Neuro2a cells but no significant inhibition of ACh-evoked Ca^2+^ influx was detected (Suppl. Figure 1).

### Selective inhibition of heteromeric human neuronal nAChRs by SLURP-1

**Figure 6:**
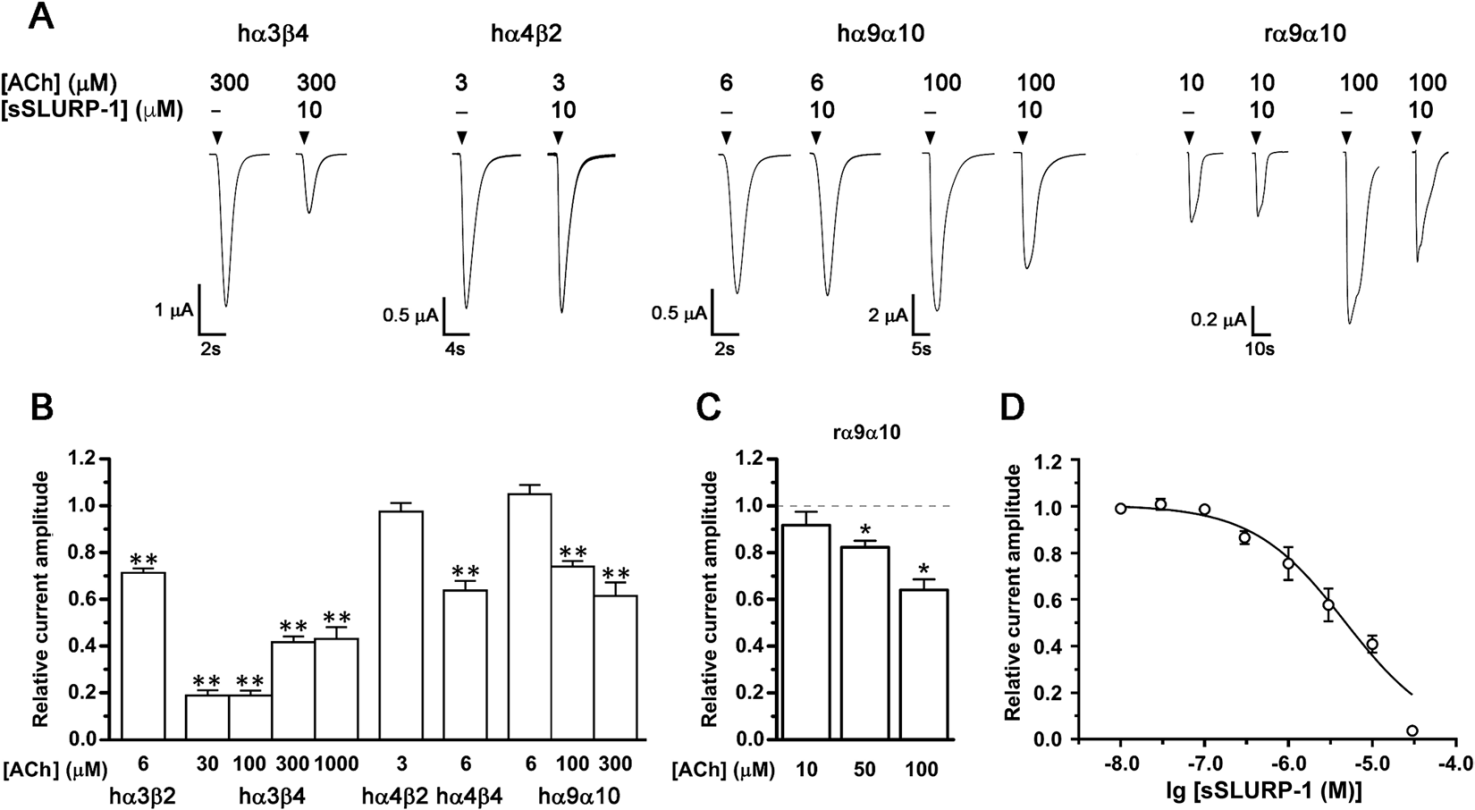
Activity of synthetic SLURP-1 on heteromeric human and rat neuronal nAChRs. (**A**) Representative ACh-evoked current traces mediated by hα3β4, hα4β2, hα9α10 and rα9α10 nAChRs in the presence of 10 µM sSLURP-1. (**B**) Bar graph of sSLURP-1 (10 μM) inhibition of ACh (EC_50_)-evoked current mediated by hα3β2 (6 µM), α3β4 (300 µM), α4β2 (3 µM), α4β4 (6 µM) and α9α10 (6 µM) nAChRs and with ACh concentrations below or above the EC_50_ for hα3β4 (30, 100 and 1000 µM) and α9α10 (100 and 300 µM). Whole-cell currents were activated by the ACh concentrations indicated. (**C**) Bar graph of 10 µM sSLURP-1 inhibition of ACh-evoked peak current amplitude mediated by rα9α10 nAChR. ACh concentrations tested were from 10 to 100 µM (ACh EC_50_ for rα9α10 receptor was 20 μM). Mean ± SEM, n = 6–14. *P < 0.05, **P < 0.0001 vs relative current amplitude in the absence of sSLURP-1, unpaired two-tailed Student’s t-test. (**D**) Concentration- response curve of sSLURP-1 inhibition of 300 µM ACh-evoked current amplitude mediated by hα3β4 nAChRs. Mean ± SEM, n = 3–10.

The activity of sSLURP-1 was also determined at respective ACh EC_50_ currents of heteromeric human nAChRs expressed in *X. laevis* oocytes (Fig. 6). sSLURP-1 at 10 µM reversibly inhibited ACh-evoked current amplitude of hα3β4 nAChRs by ~60%, whereas ~30% inhibition was observed at hα3β2 and hα4β4 nAChRs, and no inhibition was observed at hα4β2 nAChR. sSLURP-1 inhibited ACh-evoked currents mediated by hα3β4 in a concentration-dependent manner with an IC_50_ of 4.75 ± 0.78 µM (Fig. 6D). At hα3β4 nAChR in the presence of <300 µM ACh (EC_50_), sSLUPR-1 inhibition was enhanced (~80% with both 30 and 100 µM ACh), whereas with 1 mM ACh, sSLURP-1 inhibitory effect was comparable to that observed with 300 µM ACh. Interestingly, although the hα9α10 subtype was not inhibited by sSLURP-1 in the presence of 6 μM ACh (EC_50_), sSLURP-1 inhibition became clearly manifested at 100 and 300 μM ACh (~25% and ~40% inhibition of ACh-evoked current amplitude, respectively) (Fig. 6A and B). Furthermore, we also demonstrated the sensitivity of rα9α10 nAChR to sSLURP-1 inhibition which strongly correlated with the increased concentration of agonist (Fig. 6A and C).

## Discussion

Human SLURP-1 has the canonical three-finger folded structure of the snake α-neurotoxins, which are known as potent antagonists of nAChRs^4^. SLURP-1 has been shown to participate in a number of cellular regulation pathways, supposedly by acting on the homomeric α7 nAChR subtype^6–9,21,22^. However, the mechanism of interaction between SLURP-1 and α7 nAChR remains unclear due to the disparities in the activities of the recombinant human SLURP-1 constructs used. Recently, it was demonstrated that rSLURP-1 inhibited α7 nAChR-mediated currents^13^, whereas potentiation was reported previously for the myc-tagged fusion protein^12^. In contrast, binding of rSLURP-1 at the orthosteric and allosteric sites of different targets was registered^13^, which is in agreement with the similar activity of ws-Lynx1, thus supporting the proposed binding models^23,24^. We suspected these discrepancies potentially originate from the different chemical structures of the various recombinant forms of SLURP-1 used. Hence to resolve the conflicting data, we chemically synthesized the SLURP-1 protein (sSLURP-1) identical in amino acid sequence to the naturally-occurring human molecule^5^, and determined its structural and biological properties.

Three-finger proteins have been obtained previously by stepwise chemical solid phase peptide synthesis (SPPS)^25^. However, to the best of our knowledge, this approach has been limited to selected short-chain α-neurotoxins typically comprising ~60 amino acids and four disulfide bonds, with the longest being the synthetic non-conventional neurotoxin, built of 66 residues with five disulfides^26^. Larger proteins, including SLURP-1, are generally difficult to synthesize by stepwise Fmoc or Boc SPPS alone^15^.

Chemical synthesis of SLURP-1 was achieved using a convergent approach whereby the polypeptide was divided initially into three shorter peptide segments, each ranging in size of about 20–30 amino acids. The segments were prepared by Boc or Fmoc SPPS in good yield and purity, purified individually by HPLC and chemo-selectively linked together using the recently established one-pot native chemical ligation protocol^16^. Folding and disulfide bond formation of the synthetic 81-mer was achieved over 72 h using a glutathione redox shuffling system. To our knowledge, the successful chemical synthesis of SLURP-1 reported here, is the first example of a long chain three-finger protein of the Ly6 family obtained solely through chemical synthesis.

To unambiguously confirm the anticipated 3D structure, we performed NMR experiments that allowed near-complete assignment of backbone NH and CαH protons (as well as partial side chain proton assignments). This analysis and comparison with data previously obtained for rSLURP-1^19^, established that the synthetic molecule is structurally highly comparable to the recombinant protein.

Contrary to earlier reports of various recombinant SLURP-1 versions interacting with AChBPs and nAChRs^6–9,19,21^, we did not observe competition of synthetic SLURP-1 with radio-iodinated α-Bgt for binding to AChBPs of *L. stagnalis* and *A. californica*, nor to muscle-type *T. californica* nAChR (Fig. 4). Interestingly, although neither synthetic SLURP-1 and rSLURP-1 did compete with α-Bgt binding to hα7 nAChR, only rSLURP-1 inhibited ACh-evoked currents at hα7 nAChR with an IC_50_ of ~1 μM and the inhibition was enhanced with increasing ACh concentrations^13^. However, synthetic SLURP-1 did not inhibit either hα7 or rα7 nAChRs in the presence of low and high concentrations of ACh (Fig. 5A–C). The antagonistic effect of sSLURP-1 was only observed under the influence of the α7 nAChR positive allosteric modulator PNU120596, where substantial inhibition of hα7-mediated epibatidine-induced Ca^2+^ influx (Fig. 5D) was observed.

Screening of sSLURP-1 at 10 μM against a number of heteromeric human neuronal nAChR subtypes, demonstrated preferential inhibition of nAChRs co-expressing α3 and β4 subunits (Fig. 6A and B), with hα3β4 nAChR being more sensitive to inhibition by sSLURP-1 compared to hα3β2 and α4β4 nAChRs. This finding suggests that the binding site of sSLURP-1 might be located at the interface of α3 and β4 subunits and sSLURP-1 behaved as a competitive antagonist of the hα3β4 subtype. On the other hand, both hα9α10 (Fig. 6A and B) and rα9α10 (Fig. 6A and C) nAChRs showed ACh-dependent sSLURP-1 inhibition, with sSLURP-1 exerting its action at relatively high ACh concentrations. Ws-Lynx1 (a recombinant version of Lynx1 lacking the GPI anchor) was also shown to profoundly inhibit hα7, α3β2, and α4β2 nAChRs^23^, and the chimeric α7/glycine (Gly) receptor^24^ in similar fashion.

Overall, sSLURP-1 inhibition at the human αβ (except hα4β2) and α9α10 nAChRs subtypes can be registered at low ([ACh] ~EC_50_) and high ACh concentrations, respectively, conditions that are more “physiologically relevant”. In contrast, the action of sSLURP-1 on the hα7 nAChR is observed only in the presence of the artificial potentiator PNU120596.

Taken together, the results obtained for α7 and α9α10 nAChRs suggest that sSLURP-1 behaves as a ‘silent’ negative allosteric modulator, exerting its inhibitory effect at the nAChRs only when the receptor channels are in a stable open state. A similar unmasking effect on ligand activity was also reported for α-conotoxin MrIC^27^ and the marine sponge-derived 6-bromohypaphorine^28^, where both behave as an agonist by eliciting concentration-dependent increases in [Ca^2+^]_i_ via PNU120596-modified hα7 nAChR.

As for α9α10, the mode of action of sSLURP-1 on this nAChR subtype mirrors the proposed negative allosteric mechanism of rSLURP-1^13^ and ws-Lynx1^23,24^ where both are more potent inhibitors of α7 nAChR in the presence of high ACh concentrations. Despite the structural similarities in the NMR-Hα chemical shift profiles of sSLURP-1 and rSLURP-1 (Fig. 3B), these proteins clearly behave differently at their targets.

**Figure 7:**
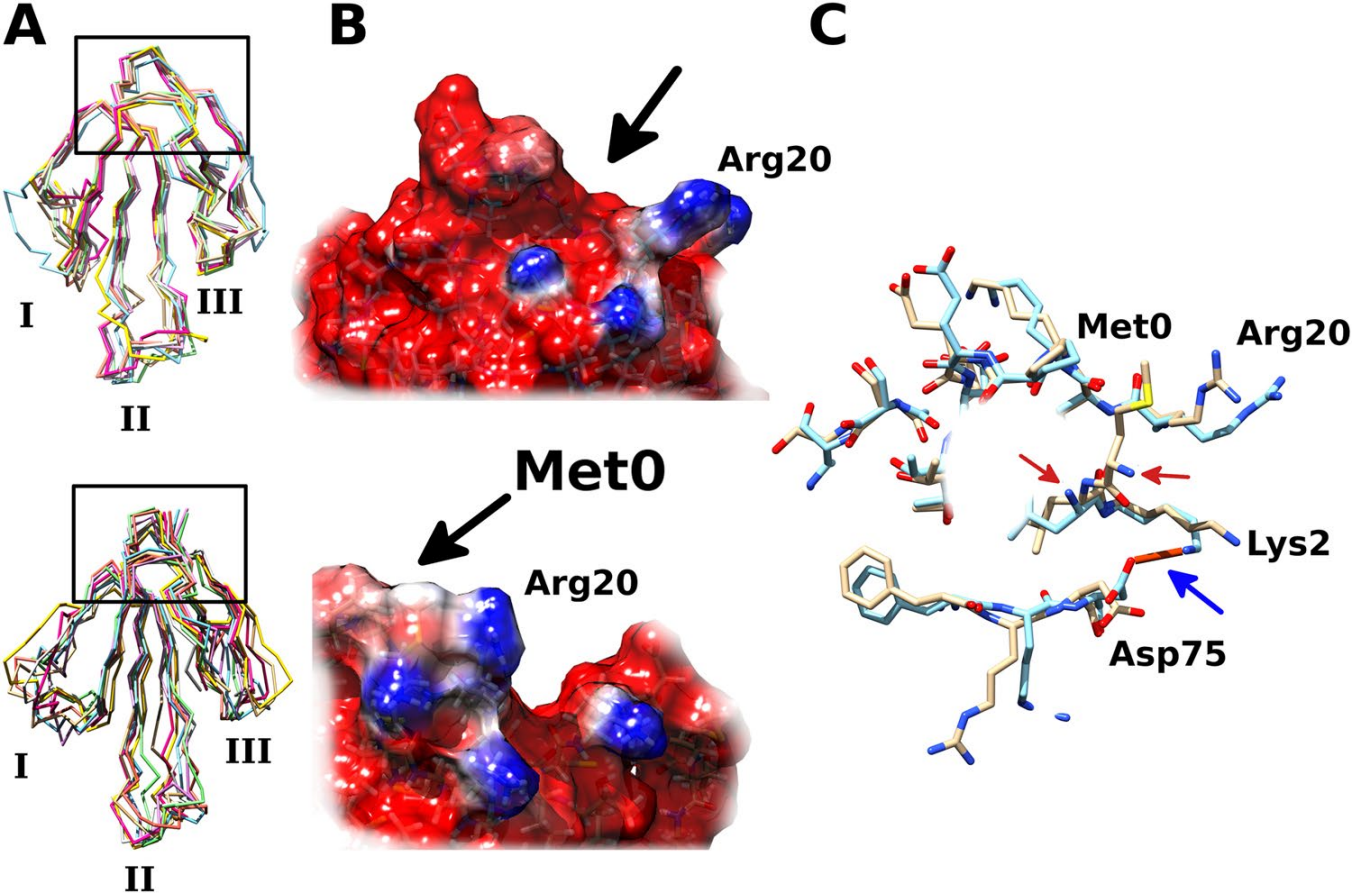
Comparison of the synthetic and recombinant human SLURP-1 structural models. (**A**) Deletion of the methionine (Met0) residue did not significantly alter the overall molecule motility of sSLURP-1 (top). For comparison, rSLURP-1 (bottom) is also presented. Seven superimposed frames from molecular dynamics simulations are shown. The N-terminal region of interest is boxed and the three protruding fingers are labeled I–III. (**B**) Electrostatic-surface profile of rSLURP-1 (bottom) and sSLURP-1 (top) showing that the positively charged Arg20 is now more solvent exposed. Arrows show regions occupied by Met0 residue in rSLURP-1 or solvent-accessible area in sSLURP-1. (**C**) Superimposed rSLURP-1 (light brown) and sSLURP-1 (cyan) structures showing the differences in the “head” region where Met0 is located in rSLURP-1. The N-terminal amino groups are indicated by red arrows. In the sSLURP-1 structure, a salt-bridge between residues Lys2 and Asp75 is present (blue arrow). Some residues have been omitted for clarity.

Using the published NMR structure of rSLURP-1 (PDB ID: 2MUO), we built a model for sSLURP-1 (Fig. 7A) to investigate in more detail the structural differences between the two proteins. Molecular dynamic simulation of both structures indicates that the additional N-terminal methionine (Met0) residue in the rSLURP-1 is tightly packed inside the disulfide-rich core of the molecule (known to be important for stabilizing the overall conformation), whereas the absence of this residue in sSLURP-1 may allow Arg20 to protrude from the protein surface, possibly allowing it to participate in receptor interactions (Fig. 7B and C). Furthermore, Lys2 and Asp75 form a stable salt-bridge, which is absent in rSLURP-1 (Fig. 7C, blue arrow). Such structural changes may account for the different biological actions observed for sSLURP-1 and rSLURP-1.

Recently, another member of the Ly6 protein superfamily, SLURP-2, was recombinantly expressed in *E. coli* with an additional N-terminal methionine residue, similar to rSLURP-1^29^. In previous publications utilizing different fusion forms, SLURP-2 was claimed to act selectively on α3-containing nAChRs^30^. However, rSLURP-2 is functionally more similar to ws-Lynx1 than to rSLURP-1. At micromolar concentrations, rSLURP-2 inhibited α4β2, α3β2 and α7 nAChRs, whereas at lower concentrations it potentiated the α7 nAChR-mediated currents^29^. This study also provided the NMR structure for rSLURP-2 (PDB ID: 2N99) revealing a considerable conformational mobility, comparable to that earlier observed for rSLURP-1. Although we did not perform a direct comparison of the various reported recombinant forms of SLURP-1, our results suggest that even one additional methionine residue at the N-terminus, probably by affecting the spatial structure, can produce marked changes in the functional activity of Ly6 proteins.

## Conclusions

We wish to emphasize that our work on the synthetic protein identical in amino acid sequence to the naturally-occurring human SLURP-1 does not undermine the previous work on Ly6 analogs produced in *E. coli*. The activities reported with them may open new avenues to diagnostics and drug development. However, our results clearly show that unraveling physiologically-relevant mechanisms for endogenous regulators requires the study of compounds which should be as close as possible to the native proteins. In this context, total chemical synthesis has been particularly instrumental in the past for small proteins, and recent advances in peptide chemistry^14,31^ have allowed this concept to be extended to larger proteins. Furthermore, our work highlights the need for strict compound characterization standards if results are to be reproducible and transferable. With the advancements in modern analytical techniques that are customary in synthetic organic chemistry (high-resolution mass spectrometry, NMR and X-ray structure determination) it has become necessary to apply the same rigorous standards to proteins and other biologics produced by recombinant expression.

Our study revealed, for the first time, human sSLURP-1 interactions with several neuronal nAChR subtypes (hα3β4, hα3β2, hα4β4 and hα9α10). We expect these findings will be important for understanding the *in vivo* function of SLURP-1 in human health and disease in the future.

## Methods

### Peptide synthesis

All amino acids used were of the L-configuration. The SLURP-1[51–81] (CSSSCVATDPDSIGAAHLIFCCFRDLCNSEL) segment was synthesized by automated Fmoc SPPS using standard protocols. The peptide was assembled on 2-chlorotrityl chloride resin using the following side chain protecting groups: Cys(Trt), Asp(tBu), Glu(tBu), His(Trt), Asn(Trt), Arg(Pbf), Ser(tBu) and Thr(tBu). Resin cleavage and side-chain deprotection were carried out by suspending the dried peptide-resin in cleavage cocktail (trifluoroacetic acid (TFA):triisopropylsilane: H_2_O;95:2.5:2.5) (v/v/v)). After stirring for 1.5 h at room temperature, majority of the TFA was evaporated under vacuum and the peptide was precipitated with ice-cold diethyl ether. The peptide was dissolved in 50% acetonitrile (ACN)/water containing 0.05% TFA and lyophilized. Peptide α-thioalkylesters corresponding to SLURP-1[1–20]- α -thioester (LKCYTCKEPMTSASCRTITR-*[COS]*-Ser) and SLURP-1[21–50]-α-thioester (Thz-KPEDTACMTTLVTVEAEYPFNQSPVVTRS-*[COS]*-Lys) were assembled by manual *in situ* neutralization Boc chemistry as described previously^32,33^. The following standard side chain protection groups were used: Cys(4-MeBzl), Arg(Tos), Asp(OcHx), Asn(Xan), Glu(OcHx), Gln(Xan), Lys(2Cl-Z), Ser(Bzl), Thr(Bzl), Tyr(Br-Z). Following chain assembly, peptides were side chain-deprotected and simultaneously cleaved from the resin by treatment with anhydrous HF containing 10% (v/v) *p*-cresol for 1 h at 0 °C. HF was evaporated under reduced pressure. The crude product was precipitated and washed with chilled diethyl ether, then dissolved in 50% (v/v) aqueous ACN containing 0.1% TFA (v/v) and lyophilized. Peptides were purified by reversed-phase high-pressure liquid chromatography (RP-HPLC) using a preparative Vydac C18 (22 × 250 mm) column on a Shimadzu Prominence platform. Crude peptides were dissolved in a 10% (v/v) ACN-water mixture containing 0.05% (v/v) TFA, before being loaded onto the column pre-equilibrated with 10% of solvent B (ACN: H_2_O:TFA; 89.5:10:0.05) in solvent A (H_2_O:TFA; 99.5:0.05). Peptides were eluted using linear gradients of solvent B in solvent A, and fractions were collected across the expected elution time. Peptide purity and identity were assessed by ESI-MS on API-2000 mass spectrometer (Applied Biosystems) and by analytical scale uHPLC on a Shimadzu Nexera system equipped with an Agilent Zorbax C18 column (1.8 μm, 2.1 × 100 mm). Fractions containing the desired product were pooled, lyophilized and stored at −20 °C.

#### One-pot native chemical ligation

Initially, 76 mg of SLURP-1[51–81] (MW: 3278.7, 23.2 µmol) and 90 mg of SLURP-1[21–50] (MW: 3545.9, 25.4 µmol) were dissolved in 15 mL of ligation buffer (6 M GdmHCl, 200 mM Na-phosphate, 50 mM mercaptophenylacetic acid, 40 mM TCEP, pH 7.0). The mixture was stirred under an argon atmosphere for 12 h after which LC-MS analysis indicated near quantitative product formation, yielding SLURP-1[21–81] (Cys21Thz) with an observed mass 6590.2 ± 0.6 Da, calculated mass: 6590.4 Da (average isotope composition).

Methoxyamine HCl was added to a final concentration of 250 mM and the pH was adjusted to 4.0–4.1 with concentrated HCl. The reaction was left for 8 h and stirred under an argon atmosphere. The pH was adjusted to 7.0 by adding 4 M NaOH and 71 mg of SLURP-1[1–20] (MW: 2467.8, 28.8 μmol) were subsequently added. The pH was re-adjusted again to 6.9–7.0, and the mixture was stirred at room temperature for 10 h under an argon atmosphere. A fresh portion of TCEP (20 mM final concentration) was added and the mixture was stirred for another 20 min. The product was then filtered and purified by HPLC on a Phenomenex C18 column (22 × 250, 5 μm, 300 Å). It yielded 142 mg (16 μmol, 69%) of the fully reduced 81-mer polypeptide (>95% purity).

#### *In vitro* protein folding and disulfide formation

For *in vitro* folding and disulfide bond formation, 25 mg (2.8 μmol) of the purified and fully reduced peptide were dissolved in 5 mL of 6 M GdmHCl to give a concentration of 5 mg/mL. Folding was carried out at 4 °C and initiated by rapid 1:40 dilution of the peptide solution with folding buffer (100 mM Tris, 2.0 M urea, 0.5 M arginine, 4 mM reduced glutathione, 1 mM oxidized glutathione, adjusted to pH 8.0 at 4 °C with conc. HCl). The reaction was left at 4 °C and stirred for 3 days after which the mixture was acidified with TFA to give a pH of ~4, filtered and purified by HPLC on a Zorbax C3 column (10 × 250, 3 μm, 300 Å). The mass of synthetic SLURP-1 was determined by high-resolution ESI-MS on an AB SCIEX 5600 Triple-TOF mass spectrometer equipped with a nanoelectrospray ionization source. SLURP-1 observed mass 8837.1 ± 0.1 Da; calculated mass 8837.02 Da (monoisotopic mass). Isolated yield: 11.4 mg (1.3 μmol, 46%).

#### NMR analysis

Synthetic SLURP-1(4 mg) was dissolved in 500 μL of 90% H_2_O/10% D_2_O solution and adjusted to pH 4.8 by adding 1 M NaOH. 2D ^1^H-^1^H TOCSY, NOESY as well as ^1^H-^15^N HSQC spectra were collected at 310 K using a 600 MHz Bruker spectrometer equipped with a cryogenically cooled probe. All spectra were recorded with an interscan delay of 1.0 s. The NOESY and TOCSY mixing times were 200 ms and 80 ms, respectively. Standard Bruker pulse sequences were used with WATERGATE for solvent suppression. NMR data were processed using Topspin (Bruker) and analyzed by CCPNMR^34^.

#### Molecular modeling

SLURP-1 was modeled by removing the N-terminal methionine residue from the PDB 2MUO structure using UCSF Chimera^35^. Both sSLURP-1 and rSLURP-1 structures were subjected to consequent equilibration 100 ns NVT (constant number of particles, volume and temperature) and 100 ns NPT (constant number of particles, pressure and temperature) ensemble simulations with constrained heavy atoms followed by 50 ns unconstrained molecular dynamics simulations using the GROMACS-5.0.4 package (reference temperature 310 K, 100 mM NaCl). Then, three individual frames from each of these two simulations were used as starting structures for 5 ns unconstrained molecular dynamics simulations to confirm reproducibility of results.

### Electrophysiology

#### In vitro cRNA synthesis

Plasmid pMXT construct of human nAChR α7 and plasmid pSP64 construct of human nAChR α4 were linearized with *BamHI*, and plasmid pT7TS constructs of human nAChR α3, α9, α10, β2, and β4 were linearized with *XbaI* restriction enzymes (NEB, Ipswich, MA, USA).

Plasmid pcDNA3.1/Hygro(+) construct of rat nAChR α7 was linearized using *XbaI*, and plasmid pSGEM constructs of rat nAChR α9 and α10 were linearized using *NheI* restriction enzymes (Promega, Madison, WI, USA). All linearized plasmid constructs were subjected to *in vitro* cRNA transcription using SP6 (human nAChR α7 and α4) and T7 (human nAChR α3, α9, α10, β2, and β4, and rat nAChR α7, α9 and α10) mMessage mMachine® transcription kits (AMBION, Foster City, CA, USA).

#### Oocyte preparation and microinjection

Stage V-VI oocytes (Dumont’s classification; 1200–1300 μm in diameter) were obtained from *Xenopus laevis*, defolliculated with 1.5 mg/mL collagenase Type II (Worthington Biochemical Corp., Lakewood, NJ, USA) at room temperature (21–24 °C) for 1–2 h in OR-2 solution containing (in mM) 82.5 NaCl, 2 KCl, 1 MgCl_2_ and 5 HEPES at pH 7.4. Oocytes were injected with 5 ng of human nAChR α3β2, α3β4, α4β2, α4β4 or α7 cRNAs, 35 ng of human nAChR α9α10 cRNA or 9 ng of rat nAChR α7 and α9α10 cRNA (concentration confirmed spectrophotometrically and by gel electrophoresis) using glass pipettes pulled from glass capillaries (3-000-203 GX, Drummond Scientific Co., Broomall, PA, USA). Oocytes were incubated at 18 °C in sterile ND96 solution composed of (in mM) 96 NaCl, 2 KCl, 1 CaCl_2_, 1 MgCl_2_ and 5 HEPES at pH 7.4, supplemented with 5% fetal bovine serum (FBS), 50 mg/L gentamicin (GIBCO, Grand Island, NY, USA) and 10000 U/mL penicillin-streptomycin (GIBCO, Grand Island, NY, USA). All procedures were approved by the University of Sydney Animal Ethics Committee and were performed in accordance with the Australian code of practice for the care and use of animals for scientific purposes (8^th^ edition, 2013).

#### Oocyte two-electrode voltage clamp recording and data analysis

Electrophysiological recordings were carried out 2–7 days post cRNA microinjection. Two-electrode voltage clamp recording of *X. laevis* oocytes expressing human nAChRs was performed at room temperature (21–24 °C) using a GeneClamp 500B amplifier and pClamp9 software interface (Molecular Devices, Sunnyvale, CA, USA) at a holding potential −80 mV. For rat nAChRs, electrophysiological recordings were made using turbo TEC-03X amplifier (NPI Electronic, Germany) and WinWCP recording software (University of Strathclyde, UK), at a holding potential −60 mV. Voltage-recording and current-injecting electrodes were pulled from GC150T-7.5 borosilicate glass (Harvard Apparatus, Holliston, MA, USA) and filled with 3 M KCl, giving resistances of 0.3–1 MΩ.

Oocytes expressing human nAChR α9α10 were incubated with 100 μM BAPTA-AM (Sigma-Aldrich, St. Louis, MO, USA) at 18 °C for ~3 h before recording and perfused with ND115 solution containing (in mM): 115 NaCl, 2.5 KCl, 1.8 CaCl_2_, and 10 HEPES at pH 7.4. Oocytes expressing rat nAChRs were perfused with Ba^2+^ Ringer’s solution containing (in mM) (115 NaCl, 2.5 KCl, 1.8 BaCl_2_, 10 HEPES at pH 7.2), whereas other human nAChR-expressing oocytes were perfused with ND96 solution. All oocytes were perfused at a rate of 2 mL/min in an OPC-1 perfusion chamber of < 20 µL volume (Automate Scientific, Berkeley, CA, USA).

Initially, oocytes were briefly washed with bath solution (ND96/ND115/Ba^2+^ Ringer’s solution) followed by 3 applications of ACh using a HPLC injector with a 50 µL sample loop. Washout with bath solution was done for 3 min between ACh applications. Oocytes were incubated with sSLURP-1 for 5 min with the perfusion system turned off, followed by co-application of ACh and sSLURP-1 with flowing bath solution. All sSLURP-1 solutions were prepared in ND96/ND115 + 0.1% bovine serum albumin (BSA), except for sSLURP-1 in Ba^2+^ Ringer’s solution. Peak current amplitudes before (ACh alone) and after (ACh + sSLURP-1) sSLURP-1 incubation were measured using Clampfit 10.7 software (Molecular Devices, Sunnyvale, CA, USA) or WinWCP software (University of Strathclyde, UK), where the ratio of ACh + sSLURP-1-evoked current amplitude to ACh alone-evoked current amplitude was used to assess the activity of sSLURP-1 at nAChRs. All electrophysiological data were pooled (n = 3 to 14) and represent means ± standard error of the mean (SEM). Data analysis was performed using GraphPad Prism 7 (GraphPad Software, La Jolla, CA, USA). Data sets were compared using unpaired two-tailed Student’s t-test. Differences were regarded statistically significant when p < 0.05. The IC_50_ was determined from concentration-response curve fitted to a non-linear regression function and reported with error of the fit.

### Calcium imaging of SLURP-1 interaction with α7 nAChR

Mouse neuroblastoma Neuro2a cells were cultured in Dulbecco’s modified Eagle’s medium (DMEM, PanEco, Russia) supplemented with 10% FBS (PAA Laboratories, Austria). Cells were sub-cultured 24 h before transfection and were plated at density of 10,000 cells per well (black 96-well plate, Corning, USA), followed by Lipofectamine (Invitrogen, USA) -mediated transient co-transfection of hα7 nAChR-pCEP4, fluorescent calcium sensor pCase12-cyto (Evrogen, Russia) and chaperone Ric3-pCMV6-XL5 or NACHO TMEM35-pCMV6-XL5 plasmid constructs (OriGene, USA). Mouse muscle α1, β1, δ and ε nAChR-pRBG4 plasmid constructs were expressed similarly, but without a chaperone.

Transfected Neuro2a cells were grown at 37 °C in 5% CO_2_-incubator for 48–72 h, then medium was removed and the cells were washed with external buffer containing (in mM) 140 NaCl, 2 CaCl_2_, 2.8 KCl, 4 MgCl_2_, 20 HEPES, 10 glucose at pH 7.4. Cells were pre-incubated with 5 μM sSLURP-1 for 20 min at room temperature before agonist addition (ACh or epibatidine (Tocris, UK)). To potentiate α7 nAChR response, PNU120596 (10 μM) was added to the pre-incubation solution. Cells were excited at 485 nm and emitted fluorescence was detected at 535 ± 10 nm, using a multimodal microplate reader Hidex Sense (Hidex, Turku, Finland). Fluorescence was recorded every 2 s for 3 min following agonist addition. Responses were measured as peak intensity minus basal fluorescence level, and are expressed as a percentage of a maximal response obtained to agonist. Data files were analyzed using HidexSence software (Hidex, Turku, Finland) and OriginPro 7.5 software (OriginLab, MA, USA, for statistical analysis). Negative controls were run in the presence of 4 μM α-Cbt.

### Radioligand assay of sSLURP-1 binding to AChBPs and nAChRs

In competition experiments with [^125^I]-α-Bgt, sSLURP-1 (1–100 μM) was pre-incubated 3 h at room temperature with AChBPs (*L. stagnalis* AChBP or *A. californica* AChBP at final concentrations of 2.4 nM, and 140 nM, respectively) or nAChRs (hα7 nAChR-expressing GH_4_C_1_ cells or *T. californica* electric organ membranes at a final concentration of toxin-binding sites of 0.4 nM and 1.25 nM, respectively (measured using [^125^I]-α-Bgt)), in 50 μL buffer consisting of 20 mM Tris-HCl and 1 mg/mL BSA, pH 8.0 (binding buffer).

Radioiodinated α-Bgt was added to a final concentration of 0.2 nM, and the mixture was incubated for 5 min. Binding was stopped by rapid filtration on double DE-81 filters (Whatman, Maidstone, UK) pre-soaked in binding buffer (for AChBPs) or GF/C filters (Whatman, Maidstone, UK) pre-soaked in 0.25% polyethylenimine (for GH_4_C_1_ cells and *T. californica* electric organ membranes), unbound radioactivity was removed from the filters by washout (3 × 3 mL) with the binding buffer. Non-specific binding was determined in all cases using 3 h pre-incubation with 10 μM α-Cbt.

### Data availability

All data generated during this study are included in the published article and the supplementary information file.

## Electronic supplementary material

Supplementary Data
