# Supplementary material for: Interaction of Synthetic Human SLURP-1 with the Nicotinic Acetylcholine Receptors
Source: Sci Rep. 2017 Nov 30;7:16606. doi: 10.1038/s41598-017-16809-0 (PMC5709491; doi:10.1038/s41598-017-16809-0)
Supplement: Supplementary file 1 — Supplementary Data [file 41598_2017_16809_MOESM1_ESM.pdf]

Supplementary Information

Interaction of Synthetic Human SLURP-1 with the Nicotinic Acetylcholine Receptors

Thomas Durek[1,*], Irina V. Shelukhina[2], Han-Shen Tae[3], Panumart Thongyoo[1,5], Ekaterina N. Spirova[2], Denis S. Kudryavtsev[2], Igor E. Kasheverov[2], Grazyna Faure[4], Pierre-Jean Corringer[4], David J. Craik[1], David J. Adams[3] and Victor I. Tsetlin[2,*]

[1]Institute for Molecular Bioscience, The University of Queensland, Brisbane, QLD 4072, Australia.
[2]Shemyakin-Ovchinnikov Institute of Bioorganic Chemistry, Russian Academy of Sciences, Moscow, 117997, Russia.
[3]Illawarra Health and Medical Research Institute (IHMRI), University of Wollongong, Wollongong, NSW 2522, Australia.
[4]Channel-Receptors Unit, Institut Pasteur, 75015 Paris, France; CNRS UMR 3571, 75015 Paris, France.
[5]present address: Faculty of Science and Technology, Thammasat University, Bangkok, Thailand
*Correspondence and requests for materials should be addressed to V.I.T. (e-mail: victortsetlin3f@gmail.com) or T.D. (e-mail: t.durek@uq.edu.au)

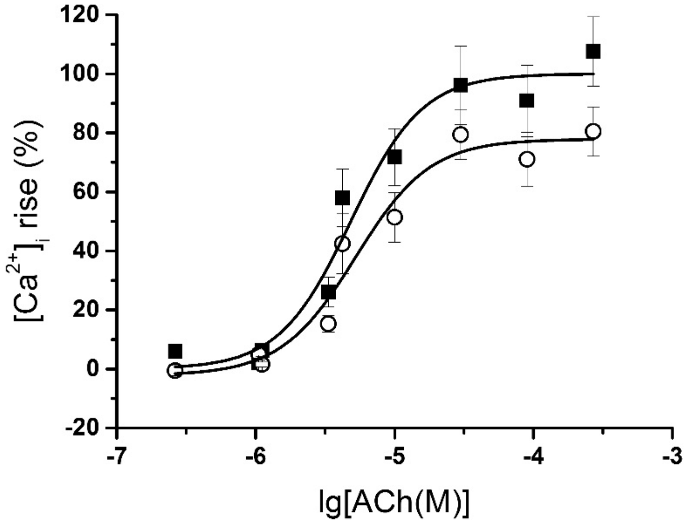

Suppl. Fig. 1: Activity of synthetic human SLURP-1 on acetylcholine (ACh)-evoked response mediated by mouse muscle nAChRs expressed in Neuro2a cells. Concentration-response curve of ACh-induced intracellular calcium ion concentration ($[Ca^{2+}]_i$) rise in the absence (*black squares,* EC_{50} = 4.8 ± 1.0 μM) and presence of 5 μM SLURP-1 (*open circles,* EC_{50} = 5.2 ± 1.5 μM). Mean ± SEM, n = 5.
